# Accuracy and reliability of feature selection by Chinese fingerprint examiners

**DOI:** 10.1080/20961790.2017.1375449

**Published:** 2017-09-20

**Authors:** Shiquan Liu, Zhongliang Mi, Glenn M. Langenburg, Jian Wu

**Affiliations:** a Institute of Evidence Law and Forensic Science, China University of Political Science and Law, Beijing, China; b Shanghai Key Laboratory of Crime Scene Evidence, Shanghai, China; c Minnesota Bureau of Criminal Apprehension, St. Paul, MN, USA; d College of Sciences, Northeastern University, Shenyang, China

**Keywords:** Forensic science, fingerprint, ACE-V, minutiae, reliability, reproducibility, feature selection

## Abstract

The selection of minutiae is a critical part of the analysis phase within the fingerprint identification methodology, known as analysis-comparison-evaluation-verification. This study assessed the accuracy and reliability of the minutiae selections of 92 Chinese fingerprint examiners during the analysis phase, absent an exemplar print, of the fingerprint identification process. Specifically, we measured the accuracy (trueness) of their annotation of minutiae, and we measured their reliability which is the reproducibility and repeatability in their annotations in one complex mark by using R software. We observed significant variation within inter- and intra-examiner annotations of the minutiae. We saw no statistically significant differences for the variability of minutiae annotations based on the participant's sex or years of experience.

## Introduction

Fingerprint analysts use a general protocol called ACE-V, an acronym that represents four phases of fingerprint examination: analysis, comparison, evaluation and verification [1,2]. Minutiae annotation is the process by which a latent print examiner selects features that will aid in his or her decision-making regarding the fingermark. The examiner determines if there are enough minutiae in the analysis phase to warrant a comparison. In the ACE-V methodology, the analysis phase is an information gathering phase and feature selection is a critical part of it. The aim of this study was to explore the accuracy and reliability of feature selection which provided information about the examination of the fingermark in the analysis phase [3–5].

## Methods and materials

A national level study was conducted with fingerprint examiners in China. During a training workshop, data from 140 participants were collected; however, only complete data were available from 92 participants that were included in this study. We recorded: the number of minutiae annotated, the position of minutiae annotated, the examiner's level of confidence (using a three point scale) for the existence of the minutiae and the number of correct and false minutiae annotated [6–8]. The accuracy of the feature selection (i.e. if a minutia was “correct” or “false”) was determined by using an exemplar of the true source of the fingermark. This exemplar was never provided to the participants during their analysis. The 92 analysts were divided into three groups based on working years of experience (Table 1).

**Table 1.** Analyst information for years of experience.

Group	Working years	*n*
1	1–5	58
2	6–10	21
3	>11	13

**Figure 1.**
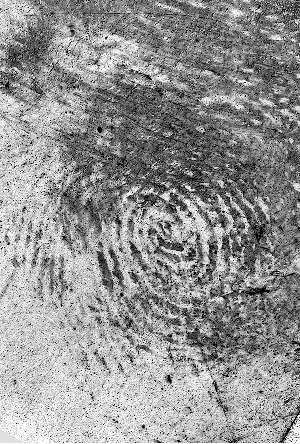
Mark 1.

The fingermark used in this study was chosen from previous research [1]. By using the same stimulus as previous research, it was possible to make a comparison between the performance of Chinese and US fingerprint examiners. Figure 1 shows the mark used in this study.

On Day 1 of the workshop, the analysts were asked to annotate the minutiae in the mark that the analyst was “confident” existed. On Day 2 of the workshop, the same analysts were asked to annotate minutiae in the same mark using a GYRO type of colour scheme. GYRO is a documentation system where different colours represent different levels of confidence by the expert regarding the existence of the chosen feature [9]. For example, in this study, minutiae that were annotated with green were indications of the examiners that they had a high level of confidence in the existence of the feature and a strong expectation to observe the feature in the exemplar print if the mark and control print share a common source.

Examiner annotations were scored for accuracy and the colour used was recorded. As a practical matter, examiners in this study actually used a blue colour to represent a moderate level of confidence (the yellow colour in GYRO) because it is easier to see than yellow. Although participants were allowed to annotate features using the GYRO colour red, those data were not included (similar to previously reported results for a similar comparison between US and Chinese examiners). The data were entered into Microsoft Excel for sorting and then statistical analyses were conducted using StatPlus Mac (v. 6.2.21) and R software (R 3.0.3).

**Figure 2.**
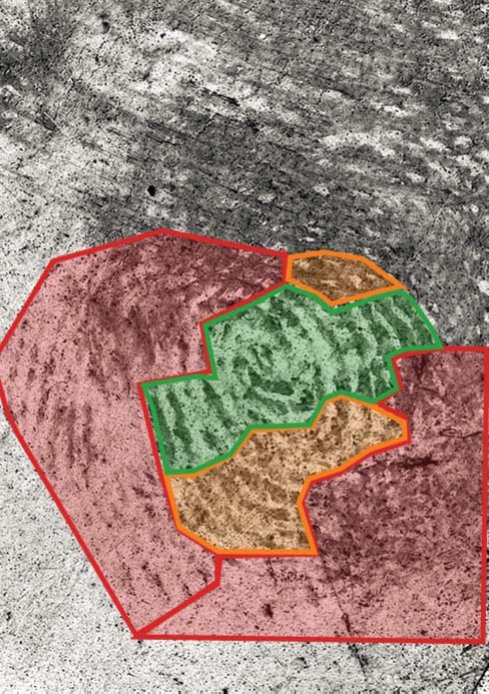
Quality area of Mark 1. The colours green, orange and red represent high, medium and low clarity, respectively.

Before the experiment, we asked fingerprint experts to mark the clarity of the mark. Examiners could mark different areas of clarity using PiAnoS software (freely available on http://ips-labs.unil.ch) [9,10]. Examiners could shade areas of the mark using the colours green, orange and red, respectively, to represent high, medium and low clarity in the area based on their experiential assessment (Figure 2). Assessing the clarity of the mark is another critical step in the analysis phase. The examiners evaluated and annotated the clarity of the mark as a means of assessing the participants’ coherence in feature selection decisions. It followed that an examiner assessing a region of high clarity would generally select features in this region with a high level of confidence, whereas regions of low clarity should have minimal features selected or features selected with a lower level of confidence.

After examiners finished the experiment, we asked a referee fingerprint expert to determine the accuracy of the participant annotations based on the ground truth minutiae from the control print associated with Mark 1 [1,9]. The “correct” and “false” minutiae were recorded for each participant.

While comparing the number of minutiae reported by an analyst can give some insight into the variability of feature selection, it does not tell the whole story. If two analysts both reported 8 minutiae, this does not mean that they had the same 8 minutiae. To truly measure reproducibility (inter-examiner variability) and repeatability (intra-examiner variability), we need to perform a deeper analysis of the repeatability of each analyst. Langenburg [1] suggested a squared Euclidean distance statistic, that he called the “minutiae variability index (MVI)”, to quantify the difference between any two analysts’ annotations (inter-examiner variability) or between the annotations of one analyst at two different times for the same mark (intra-examiner variability). This study also used MVI to quantify the stability of analyst feature selection between Day 1 and Day 2 annotations. There are four cases in calculating the MVI,

Case 1: Select correct first minutia.
Case 2: Select wrong first minutia.
Case 3: Select correct second minutia.
Case 4: Select wrong second minutia.

We treat each case equally and assign the same weight of the four cases in the MVI calculation. We define the MVI metric as

$$MVI=\sum_{i=1}^{4}w_{i}n_{i},$$

$n_{i}$ represents the number of *i* cases༌and $w_{i}$ is the weight. We gave the same weight of 1 to all cases. We are only concerned with measuring how different were the specific annotations from Day 1 to Day 2. For example, we calculated an analyst MVI as shown in Table 2. On Day 1, he or she annotated 11 minutiae including 9 correct minutiae (3, 5, 6, 7, 9, 10, 13, 17 and 18) and 2 false minutiae (30 and 32). On Day 2, he or she also annotated 11 minutiae but they were not the same 11 minutiae. This time, he or she had 8 correct ones (3, 5, 6, 7, 10, 13, 18 and 22) and 3 false ones (24, 30 and 83). We first calculated correct minutiae MVI. One correct minutiae (22) was added and two correct minutiae (9 and 17) were reduced, thus there was a change in 3 correct minutiae overall. The MVI for correct minutiae was 3. The same calculation of MVI for false minutiae was 3 (24, 32 and 83). Thus, the total MVI is 6 for this analyst even though he or she annotated the same total number of minutiae at different times.

**Table 2.** Minutiae annotated in the same mark by the same analyst at different times.

Minutiae ID	First time	Second time	Minutiae ID	First time	Second time
1	0	0	26	0	0
2	0	0	91	0	0
3	1	1	33	0	0
4	0	0	30	1	1
5	1	1	14	0	0
6	1	1	27	0	0
17	1	1	15	0	0
18	1	1	23	0	0
7	1	1	21	0	0
8	0	0	16	0	0
9	1	1	24	0	1
10	1	1	83	0	1
19	0	0	50	0	0
12	0	0	32	1	0
13	1	1	31	0	0
22	0	0	40	0	0
11	0	0	20	0	0
82	0	0	43	0	0
84	0	0	38	0	0
28	0	0	90	0	0
29	0	0	39	0	0

Student's *t* is a statistical hypothesis test in which the test statistic follows a Student's *t*-distribution under the null hypothesis to test the difference between the two results from Day 1 and Day 2. Analysis of variance (ANOVA) is a collection of statistical models used to analyse the differences among group means and their associated procedures. Differences in performance by years of experience were performed by ANOVA tests. *T*-statistic is the ratio of the departure of the estimated value of a parameter from its hypothesized value to its standard error. It was used in estimating the mean total minutiae annotated in the experiment. *F*-statistics describe the statistically expected level of heterozygosity in a population and was used to estimate the difference for the means for the three groups of experience. For all statistical texts, probabilities of less than 0.05 were accepted as significant.

## Results and discussion

### Day 1 annotation results (no GYRO)

**Figure 3.**
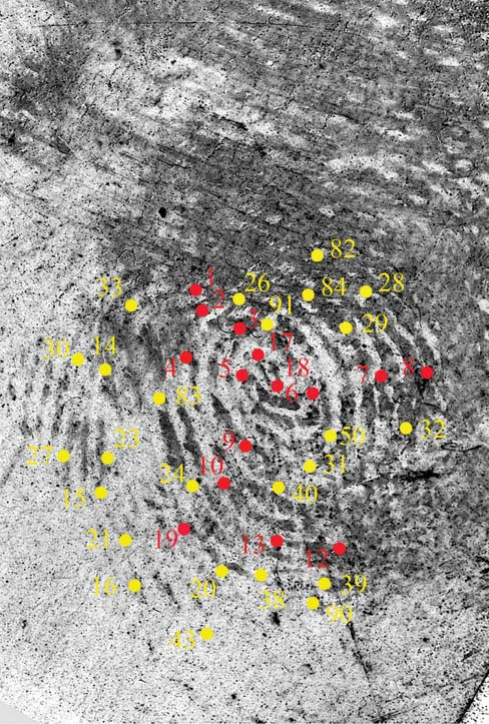
A visual graphic displaying all of the minutiae selected by at least one analyst in Mark 1. Red dots represent true/correct minutiae; yellow dots represent false/incorrect minutiae that do not correspond to a feature in the ground truth control print.

For the Day 1 annotations, there were significant differences in the total number of minutiae annotated between participants (inter-expert variability). Comparing with the control print, we scored all of the minutiae as either “correct” or “false”. A minutia was deemed “correct” if it corresponded to a true minutiae event in the same location of the control print. It was deemed “false” if no minutia was in the same location of the control print. Figure 3 is a graphical representation of all the minutiae that were annotated at least once. In Figure 3, the red coloured dots represent the correct minutiae and the yellow coloured dots represent the false minutiae.

**Figure 4.**
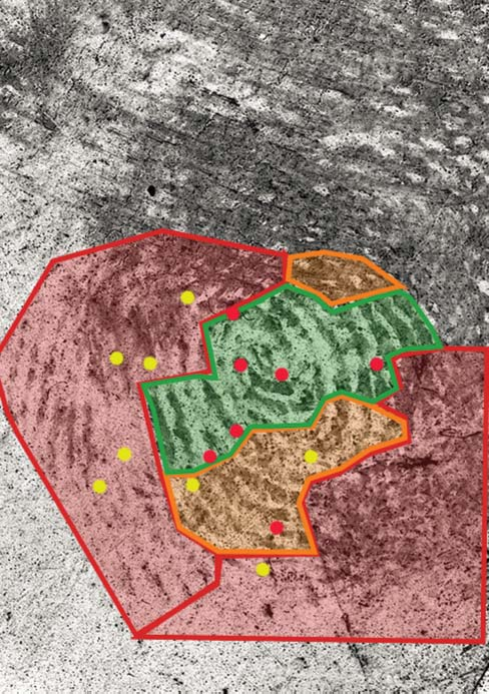
The most commonly selected correct minutiae (red) and false minutia (yellow) located in different regions of clarity.

All of the minutiae annotations (correct and false) were given a numerical designation and were recorded in Excel. The correct minutiae, per the ground truth of the control print, were designated numbers: 1, 2, 3, 4, 5, 6, 7, 8, 9, 10, 11, 12, 13, 17, 18, 19 and 22. These are the red coloured dots in Figure 3. The yellow coloured dots in Figure 3 represent the false minutiae, which were designated the following numbers: 14, 15, 16, 20, 21, 23, 24, 26, 27, 28, 29, 30, 31, 32, 33, 38, 39, 40, 43, 50, 82, 83, 84, 90 and 91. There were 17 different true minutiae and 25 different false minutiae that were selected by the participating examiners. They are located in different clarity regions (Figure 3). The data showed that 7 correct minutiae were selected by over 40% of the participants. These minutiae were designated numbers 3, 5, 6, 7, 9, 10 and 13. Figure 4 showed that 6 of these 7 consensus minutiae were located in the high clarity region (green shaded region by the examiners) and 1 of these 7 consensus minutiae was located in the medium clarity region (orange shaded area). Eight (8) false minutiae were also annotated by over 40% of the participants. These minutiae were designated as 14, 15, 20, 23, 24, 30, 31and 33. Figure 4 showed that 6 of the 8 consensus false minutiae were located in the low clarity region and the remaining 2 were located in the medium clarity region.

### Day 2 annotation results

**Figure 5.**
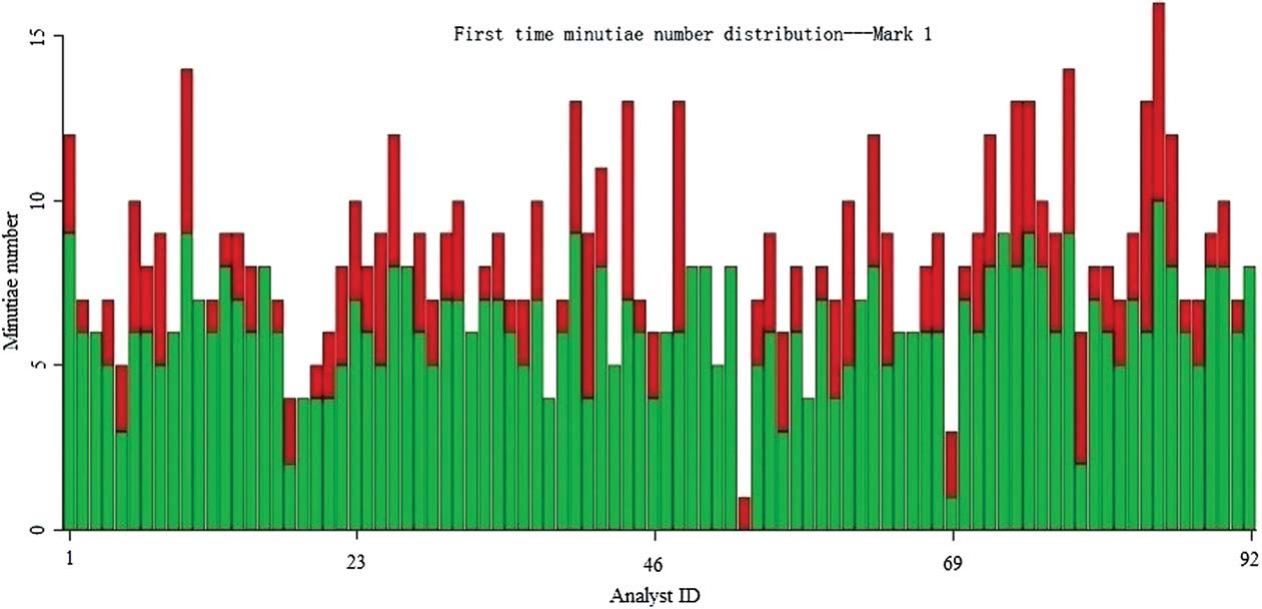
Number of minutiae annotated by each analyst on Day 1 (green represents correct minutiae, red represents false minutiae). The *X*-axis: each individual analyst, *Y*-axis: total number of minutiae that each analyst annotated (correct + false = total minutiae).

**Figure 6.**
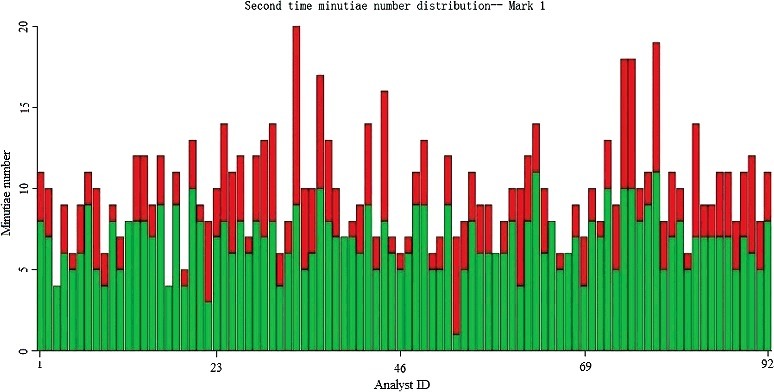
Number of green and yellow minutiae annotated by each analyst on Day 2 (green represents correct minutiae, red represents false minutiae). The *X*-axis: each individual analyst, *Y*-axis: total number of minutiae that each analyst annotated (correct + false = total minutiae).

**Figure 7.**
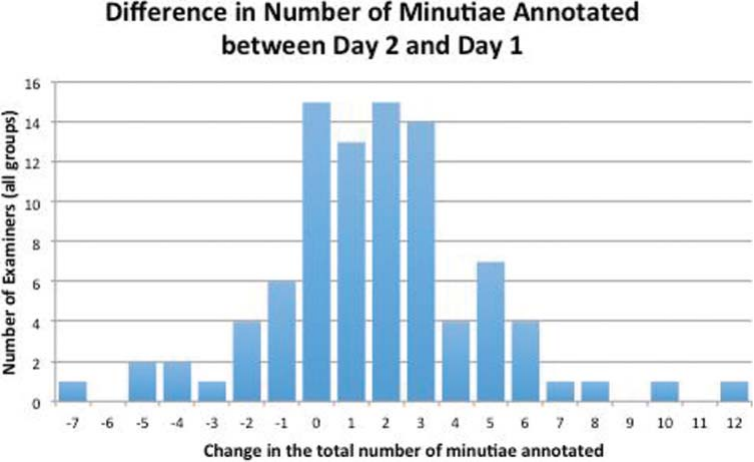
Histogram showing the change in the total number of minutiae annotated by each participant (*N* = 92) between Day 1 and Day 2 (Day 2 minus Day 1).

Figures 5 and 6 show the number of minutiae annotated by each of the 92 participants from Day 1 and Day 2. Figure 7 shows the difference (total minutiae annotated on Day 2 minus the total minutiae annotated on Day 1) between the number of minutiae annotated on Day 1 and Day 2. Participants generally increased the number of minutiae annotated by 2 minutiae on Day 2 (median value = 2).

We compared the two results from Day 1 and Day 2 for significant differences using the student *t*-test. The results showed that there was a statistically significant difference (*t*-statistic = −5.319; *P* < 0.001) in the total number of minutiae marked by each analyst between the two annotation times. The Day 2 examiners had an average 1.7 more minutiae than they had annotated on Day 1. The total number of minutiae annotated increased from 762 on Day 1 to 917 on Day 2. On the surface, there appeared to be an improvement in the volume of features selected on Day 2.

However, this increase in the number of minutiae annotated between Days 1 and 2 does not tell the entire story. Further analysis showed that participants annotated 566 total correct minutiae and 196 total false minutiae on Day 1 (mean = 6.2 correct minutiae (SD = 1.8) and 2.1 false minutiae (SD = 2.1) per analyst). On Day 2, using GYRO (considering only green and yellow, higher confidence features), participants annotated more features. Participants annotated 628 total correct minutiae and 289 total false minutiae (mean = 6.8 correct minutiae (SD = 1.9) and 3.1 false minutiae (SD = 3.1) per analyst). Thus, analysts annotated more correct minutiae in the second time, but simultaneously, increased the total number of false minutiae in the second time. This is likely due to the GYRO annotation system allowing for lower confidence features to be selected.

In selecting lower confidence features, participants did actually select additional correct minutiae, but at the cost of selecting additional false minutiae. Depending on the agency's policy and consequences for the selection of false minutiae during the analysis phase, the benefits of selecting lower confidence minutiae are debatable. For example, as noted by Langenburg [1], US examiners rarely have a penalty for the selection of false minutiae in the analysis phase. They can discount those false minutiae and attribute them to distortion artefacts during the comparison phase. Dutch examiners on the other hand are held accountable for false minutiae and are required to address them in a formal manner and cannot dismiss them so quickly. This difference in accountability led to noticeable differences in minutiae selection between Dutch and US examiners. Based on the results of this study, it appears that Chinese examiners may more closely mimic US examiners in this philosophy, given the large inter-expert variability and high proportion of false minutiae present in these data.

### Inter-expert variability differences from sex or years of experience

We explored whether there was a statistically significant difference in the mean total minutiae annotated, the number of false minutiae annotated between male and female participants, and the MVI for intra-examiner markings. Table 3 below shows critical statistics for these comparisons. Significance at the 0.05 level occurred, as indicated by the *P*-value, if a *t*-statistic was greater than the critical *t*-statistic 1.98. A student *t*-test was used to compare male and female results. No statistically significant differences in means were observed. These data are similar to findings reported elsewhere [1]. Langenburg noted that males in one experiment tended to report minutiae totals equal to, or slightly higher (but not statistically significant due to high variance) than females. We found the same in this paper.

**Table 3.** Critical statistics for the comparison of annotations for male and female analysts.

Items	Sex	*n*	Mean ± SD	*t*-statistic	*P*-value
Total minutiae for Day 1	M F	61 29	8.4 ± 2.7 8.2 ± 2.5	0.396	0.693
Total minutiae for Day 2	M F	61 29	10.0 ± 3.0 10.0 ± 3.8	0.094	0.925
Number of false minutiae Day 1	M F	61 29	2.4 ± 1.8 1.7 ± 1.6	1.624	0.108
Number of false minutiae Day 2	M F	61 29	3.2 ± 1.8 3.1 ± 2.7	0.121	0.904
MVI Intra-examiner annotations	M F	61 29	6.9 ± 2.7 6.1 ± 2.9	1.315	0.192

M: male; F: Female; there were two participants who did not answer the question regarding their sex.

We also explored differences in performance by years of experience (see Table 1 for demographics). We performed ANOVA tests for the three experience groups, comparing means for total number of minutiae, number of false minutiae and MVI intra-examiner differences between Day 1 and 2. Table 4 shows the critical statistics for these tests. No statistically significant differences were observed among the means for the three groups of experience; the critical *F*-statistics was 3.10 at the 0.05 level of significance. These results are in accordance with previously reported results [1].

**Table 4.** ANOVA tests for significant differences in minutiae annotations among three groups of experience.

Items	Group	*n*	Mean ± SD	*F*-statistics	*P*-value
Total minutiae for Day 1	1 2 3	13 21 58	9.3 ± 2.8 9.0 ± 2.7 7.8 ± 2.8	2.642	0.077
Total minutiae for Day 2	1 2 3	13 21 58	10.1 ± 2.1 10.7 ± 3.8 9.7 ± 2.8	0.716	0.492
Number of false minutiae Day 1	1 2 3	13 21 58	2.4 ± 2.1 2.6 ± 1.7 1.9 ± 2.8	1.490	0.231
Number of false minutiae Day 2	1 2 3	13 21 58	3.5 ± 1.7 3.2 ± 2.4 3.0 ± 2.8	0.234	0.792
MVI Intra-examiner annotations	1 2 3	13 21 58	6.2 ± 2.8 6.8 ± 2.6 6.6 ± 2.8	0.145	0.865

Group 1: 1 to 5 years; Group 2: 6 to 10 years; Group 3: >11 years.

From these data, we see no effects on the accuracy or variability of the minutiae selection based on the sex or experience of the Chinese examiners.

### Reliability of feature selection

For the three groups of examiners, Groups 1, 2 and 3, separated respectively by low (1–5 years of experience), moderate (6–10 years) and high (>11 years) experience, the frequency of specific annotated minutiae was recorded in Table 5. For example, a feature coded “red” was marked by 85% to 100% of the participants (a very high consensus regarding that specific feature). However, a feature coded “green” has a rate of reproducibility of annotations – only between 20% and 50%. It is important to note that some of the minutiae in Table 5 included some false minutiae as well. Some false minutiae were commonly marked by examiners.

**Table 5.** Frequency of specific annotated minutiae by experience group.

	Group 1		Group 2		Group 3	
Colour	Minutiae #	Total number	Minutiae #	Total number	Minutiae #	Total number
Red (85%–100%)	7,10	2	5,7,10	3	5,7,9,10	4
Purple (70%–85%)	5,6,9	3	6	1	6,13	2
Blue (50%–70%)	3,13	2	3,9,13	3	3,19	2
Green (20%–50%)	4,14,20,24	4	18,8,19,26,30,23,24,31,20	9	2,8,14,15,23,30,43	7

Based on the data shown in Tables 5 and 6, there are no obvious differences for the correct minutiae annotated by over 70% participants. Even Group 1annotated the same minutiae as annotated by Group 3. The only difference was a higher consensus of annotations in Group 3. The same 6 minutiae annotated by over 70% of the experienced examiners were also annotated by the lower experienced examiners, but at a lower rate of consensus. This suggests that the ability to correctly select features in high quality area is the same in the three groups and does not improve with more years of experience, but consensus appeared to be higher (although marginally so) in the more experienced group.

**Table 6.** Total number and error rates of specific annotated minutiae by experience group.

Items	Group 1	Group 2	Group 3
Total number (false + correct)	11(3+8)	16(6+10)	15(5+10)
Error rates ((false minutiae/total minutiae) × 100%)	27.2%	37.5%	33.3%

**Figure 8.**
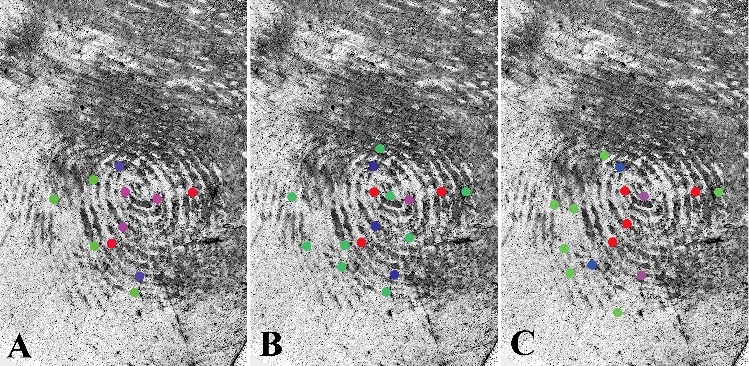
Frequency maps of three groups (red: 85%–100%; purple: 70%–85%; blue: 50%–70%; green: 20%–50%). (A) Group 1; (B) Group 2; (C) Group 3.

From Figure 8, we can see that Group 1 selected fewer minutiae in low clarity areas than Groups 2 and 3. As a result, Group 1 has a lower error rate of minutiae annotation. Groups 2 and 3 had more consensus minutiae (16 and 15, respectively) compared to Group 1 (11). However, Groups 2 and 3 had more consensus false minutiae (6 and 5, respectively) compared to Group 1 (3). Comparing the three groups, Group 2 and 3 seemed to engage in riskier behaviour when selecting minutiae in low clarity regions. As a result, they reported more correct consensus minutiae, but also reported more incorrect false minutiae. As noted earlier, this behaviour may result in consequences depending on agency approach, or potentially lead to erroneous conclusions.

## Conclusion

Similar to other studies [1,11,12], we observed significant variability for Chinese fingerprint examiners in both inter-expert and intra-expert annotations of minutiae annotations during the analysis of a complex mark.

(1) Regarding the accuracy of minutiae selection, Chinese examiners were more accurate when selecting minutiae in high clarity areas of the mark and had higher error rates when attempting to select minutiae in lower clarity regions of the mark. Minutiae selected in high clarity areas tend to be accurate, but the minutiae selected in low clarity areas should be considered carefully and sceptically. Using a standardized annotation procedure and including a more transparent mechanism for assigning analyst uncertainty during feature selection (i.e. high confidence and moderate confidence designations) appeared to improve the accuracy of minutiae selection and may lead to further understanding of how examiners select ridge characteristics.
(2) Similar to US examiners, Chinese examiners exhibited significant inter- and intra-expert variability. Although more correct minutiae were selected when using GYRO annotation system, this came at the cost of also selecting more false minutiae.
(3) There were no statistically significant differences (student *t*-test and ANOVA) when comparing the mean number of minutiae selected, mean minutiae variability index and mean number of false minutiae for three groups of experience (low, moderate and high) and for male and female Chinese examiners.
(4) The more experienced groups of examiners showed the highest consensus rate for accurate minutiae. More experienced examiners appeared to engage in riskier behaviour by selecting more features in lower quality regions. This resulted in more correct minutiae being selected, but at the cost of annotating more false minutiae.

The results showed that Chinese fingerprint examiners make feature selection choices very similarly to US examiners. Even in China, where a 12-point numerical threshold is enforced, this does not appear to impact feature selection accuracy or variability. We offer that a standardized feature selection process, coupled with technology, such as regional quality mapping [13], may reduce variability and set rules for the selection of features.
